# Real-time identification of two substrate-binding intermediates for the light-driven sodium pump rhodopsin

**DOI:** 10.1016/j.jbc.2021.100792

**Published:** 2021-05-18

**Authors:** Tomoya Kato, Takashi Tsukamoto, Makoto Demura, Takashi Kikukawa

**Affiliations:** 1 Graduate School of Life Science, Hokkaido University, Sapporo, Japan; 2 Faculty of Advanced Life Science, Hokkaido University, Sapporo, Japan; 3 Global Station for Soft Matter, Global Institution for Collaborative Research and Education, Hokkaido University, Sapporo, Japan

**Keywords:** bioenergetics, membrane transport, photobiology, rhodopsin, sodium transport, transporter, sodium pump, retinal proteins, CP, cytoplasmic, EC, extracellular, KR2, *Krokinobacter eikastus* rhodopsin 2, MSP, membrane scaffold protein, NaR, Na^+^-pump rhodopsin, SG, skewed Gaussian

## Abstract

Membrane transport proteins undergo critical conformational changes during substrate uptake and release, as the substrate-binding site is believed to switch its accessibility from one side of the membrane to the other. Thus, at least two substrate-binding intermediates should appear during the process, that is, after uptake and before the release of the substrate. However, this view has not been verified for most transporters because of the difficulty in detecting short-lived intermediates. Here, we report real-time identification of these intermediates for the light-driven outward current-generating Na^+^-pump rhodopsin. We triggered the transport cycle of Na^+^-pump rhodopsin using a short laser pulse, and subsequent formation and decay of various intermediates was detected by time-resolved measurements of absorption changes. We used this method to analyze transport reactions and elucidated the sequential formation of the Na^+^-binding intermediates O1 and O2. Both intermediates exhibited red-shifted absorption spectra and generated transient equilibria with short-wavelength intermediates. The equilibria commonly shifted toward O1 and O2 with increasing Na^+^ concentration, indicating that Na^+^ is bound to these intermediates. However, these equilibria were formed independently; O1 reached equilibrium with preceding intermediates, indicating Na^+^ uptake on the cytoplasmic side. In contrast, O2 reached equilibrium with subsequent intermediates, indicating Na^+^ release on the extracellular side. Thus, there is an irreversible switch in “accessibility” during the O1 to O2 transition, which could represent one of the key processes governing unidirectional Na^+^ transport.

Membrane transport proteins play vital roles by transporting various substances across cell membranes. The function “transportation” seems to be simple but needs strategic conformational changes. In particular, during substrate uptake and release, essential changes should occur as proposed by the alternating access model (1). For substrate uptake, the protein lumen should be exposed only to the relevant side of the membrane. After substrate uptake, “accessibility switching” occurs so that the protein lumen is exposed to the other side of the membrane for substrate release. Thus, the transport cycle should contain at least two substrate-binding intermediates, both of which involve the substrate but have significantly different conformations. These consecutively appearing intermediates have been successfully identified by computational analyses for some transport proteins (2, 3). However, experimental identifications are not achieved for most proteins, reflecting the difficulty in detecting and characterizing short-lived intermediates. Here, we report the real-time identification of two substrate-binding intermediates for the light-driven Na^+^-pump rhodopsin (NaR).

NaR belongs to the microbial rhodopsin family, a huge family of photoactive membrane proteins in unicellular microorganisms. Similar to visual rhodopsin in animal eyes, microbial rhodopsins consist of seven transmembrane helices and the chromophore retinal, which binds to a specific Lys residue *via* a protonated Schiff base (4, 5, 6). Upon light absorption, retinal isomerizes from the all-*trans* to the 13-*cis* state and distorts the protein conformation. This energized state of the protein is thermally relaxed to the original state *via* a variety of intermediates. During this cyclic reaction, called a photocycle, microbial rhodopsins perform various functions, such as ion pumps, ion channels, light sensors, and enzymes. The respective intermediates have their own “colors,” reflecting the differences in retinal configuration and its environment. This color difference is advantageous for analyzing photoreactions. When we use a laser pulse, a large amount of microbial rhodopsin simultaneously starts the photocycle. Thus, subsequent formations and decays of various intermediates can be detected by time-resolved measurements of the absorption changes. We adopted this method to analyze the NaR photocycle.

NaR is the third ion pump rhodopsin widely spread in marine bacteria (7, 8, 9). Unlike H^+^ and Cl^−^ pump rhodopsins, unphotolyzed NaR does not involve the substrate Na^+^. After the photocycle start, NaR captures Na^+^ at the cytoplasmic (CP) side and releases it at the extracellular (EC) side, as illustrated in Figure 1*A*. The major intermediates that appeared in the NaR photocycle are shown in Figure 1*B* (8, 10). Previously, we experimentally proved that Na^+^ uptake and release occur during the formation and decay of the O intermediate, which appears at the longer wavelength region in the latter half of the photocycle (11). In that study, we demonstrated well-matched time courses of two flash-induced signals, *viz.*, Na^+^ concentration change because of Na^+^ uptake and release by NaR and absorbance change reflecting O intermediate accumulation. However, as mentioned previously, at least two O intermediates should exist involving Na^+^ inside the protein. We previously suggested the formation of two O intermediates based on the absorbance changes but did not perform a definitive analysis (12). In the present study, we examined the NaR photocycle in detail by flash-induced absorbance changes to unravel the O intermediate substrates. Our analysis revealed that the photocycle consists of seven intermediates, including two O intermediates, O1 and O2. These O intermediates appear sequentially in the longer wavelength region and independently attain transient equilibria with other intermediates, where O1 equilibrates with the preceding intermediates, but O2 does so with the subsequent intermediate. These results indicate that accessibility switching occurs during the O1 to O2 transition.

**Figure 1:**
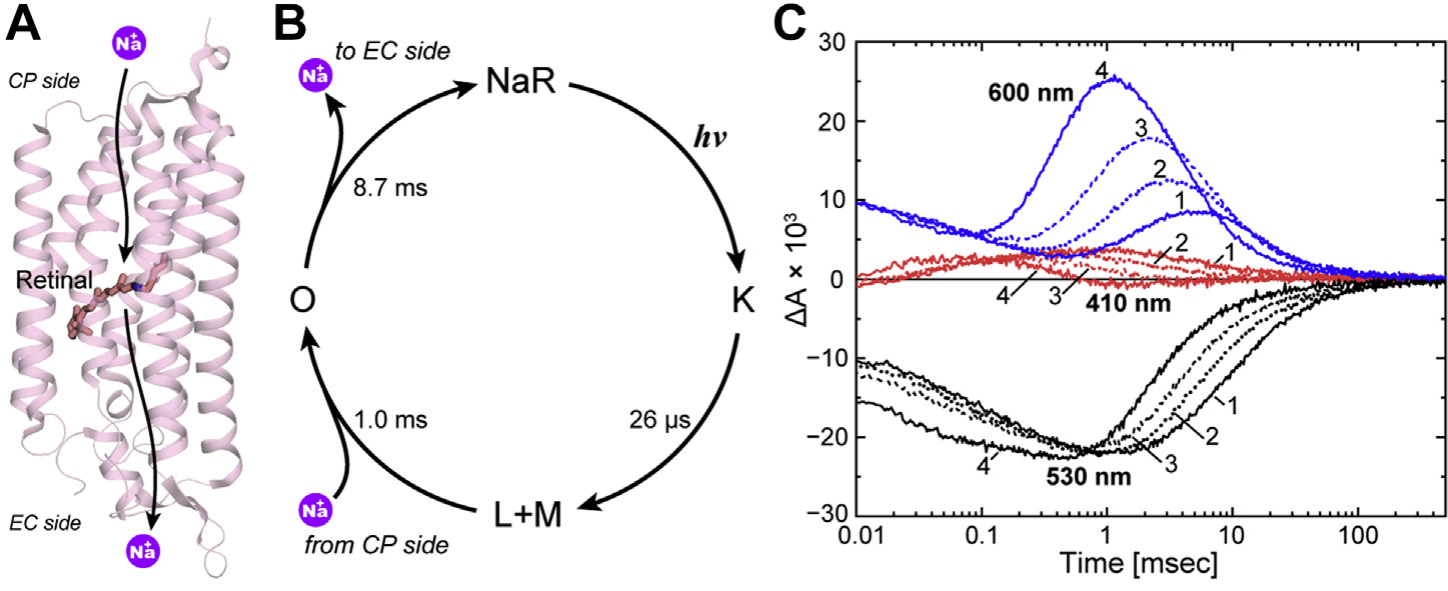
**Overview of the Na**^**+**^ **translocation of NaR.***A*, crystal structure of NaR (Protein Data Bank code: 3X3C). The photolyzed NaR captures Na^+^ at the CP side and then releases it at the EC side. *B*, a typical photocycle of NaR proposed by previous studies (8, 10, 11). The “L + M” denotes the equilibrium of L and M intermediates. They appear and decay simultaneously. *C*, flash-induced absorbance changes at three typical wavelengths: 530 nm (*black*), 410 nm (*red*), and 600 nm (*blue*). Traces 1 to 4 represent the data at 10, 30, 100, 500 mM Na^+^. The *blue traces* for 600 nm take positive values at approximately 0.01 ms, which reflect the accumulation of K. Concomitant with the decay of K, the absorbance at 410 nm (*red traces*) increases, reflecting the accumulation of the L and M mixture. Their simultaneous decay leads to O formation at 600 nm, whose accumulation reaches a maximum at approximately 1 to 10 ms. The decay of O results in the recovery of the original state, corresponding to the final increase of the *black traces* for 530 nm. CP, cytoplasmic; EC, extracellular; NaR, Na^+^-pump rhodopsin.

## Results and discussion

### Overview of the NaR photocycle

For all experiments, we used a nanodisc containing NaR to increase the clarity of the sample. Figure 1*C* shows the flash-induced absorbance changes at three typical wavelengths. The Na^+^ concentration increased from traces 1 to 4, whose respective concentrations were 10, 30, 100, and 500 mM Na^+^, respectively. The negative deflection at 530 nm represents the original dark state's depletion, whereas the positive deflection at other wavelengths indicates intermediate formation. The first intermediate K appears at a very fast rate. Thus, our apparatus followed the photocycle after the completion of K formation. The traces for 600 nm start with positive values (at 0.01 ms), which reflect the presence of remaining K. Concomitant to its decay, the short-wavelength intermediate appears at 410 nm. This absorption change was previously assigned to the equilibrium of the L and M intermediates (8, 12, 13, 14). Finally, the red-shifted intermediate O appears at 600 nm and peaks at approximately 1 to 10 ms. As described previously, the substrate Na^+^ is captured and released during the formation and decay of O. Reflecting its Na^+^-binding state, O is not formed in the absence of Na^+^ (Fig. S2), whereas in the presence of Na^+^, O accumulation becomes prominent with increasing Na^+^ concentration (Fig. 1*C*). During Na^+^ uptake and release, O should make transient equilibria with its preceding and subsequent intermediates in a manner dependent on Na^+^ concentration. To clarify these aspects and the presence of O substrates, we analyzed the photocycle in detail.

### Kinetically distinguishable states involved in the NaR photocycle

**Figure 2:**
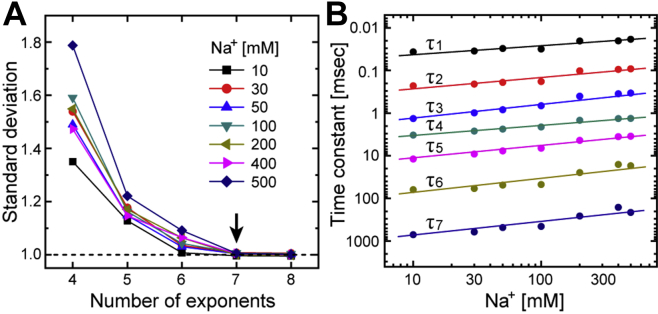
**Determination of the number of kinetically distinguishable states and their decay time constants.***A*, the standard deviations of weighted residuals are plotted against the number of exponents used for the global fitting analysis. The respective Na^+^ concentrations are indicated in the panel. Reductions in the standard deviations were saturated at seven exponents at all Na^+^ concentrations. *B*, the decay time constants of Pi states (τi) derived from a seven-exponential global fit are plotted against the Na^+^ concentration. *Straight lines* are drawn to guide the eye. The decay time constants are well separated and show monotonous changes with increasing Na^+^ concentration.

We performed global-fitting analyses for the datasets of the flash-induced absorbance changes (380–700 nm, 10 nm intervals) according to the sequential irreversible model: P0 → P1 → P2 → … → Pn → P0 (15). P0 denotes the original dark state, whereas Pi (*i* = 1, 2, … n) denotes the kinetically distinguishable states, which are not “true” intermediates. For many rhodopsins, several “true” intermediates are known to be connected with not only forward reactions but also reverse reactions. During the photocycle, these intermediates might reach quasi-equilibrium. In this case, individual intermediates are not kinetically distinguishable, but the equilibrium state is kinetically distinguishable. Consequently, these intermediates are involved in the same Pi state. Thus, the sequential irreversible model is the hypothetical model to probe the appearance order of “true” intermediates involved in the photocycle and the existence of reverse reactions (for details of analysis procedures, see Experimental procedures section). The number of P states “n” corresponds to the number of exponents in the multiexponential function used for the fitting analysis. Thus, we first performed fitting analyses by varying the number of exponents to determine the value of “n.” Standard deviations of the weighted residuals for the fitting analyses are shown in Figure 2*A*. As shown here, the decrease in standard deviations became saturated at seven exponents in the presence of any Na^+^ concentration. The number “seven” is relatively large compared with those for other microbial rhodopsins (16, 17, 18, 19). However, the validity of the seven exponents is supported by Figure 2*B*, which indicates the excellent separation of the determined decay time constants of P states (τ_i_) and their monotonous changes with increasing Na^+^ concentration. For example, the decay time constants at 200 mM Na^+^ were 20 μs, 0.10 ms, 0.40 ms, 1.5 ms, 4.3 ms, 25 ms, and 260 ms for the P1–P7 states, respectively. Thus, we concluded that the NaR photocycle involves seven P states.

**Figure 3:**
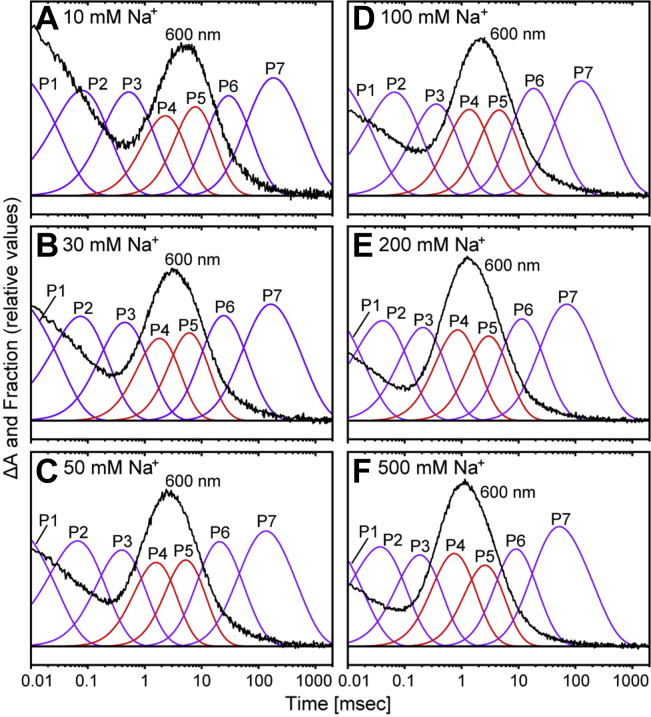
**Kinetically distinguishable states contributing to the accumulation of the O intermediate.** Time-dependent concentration changes of Pi states (*i* = 1–7) were calculated using their decay time constants derived from the global fitting analyses. These concentration changes (*purple* and *red traces*) are plotted together with the time courses of flash-induced absorbance changes at 600 nm (*black traces*) by adjusting the *y*-axes. The Na^+^ concentrations of panels (*A−F*) are 10, 30, 50, 100, 200, and 500 mM, respectively. The absorbance changes resulted in peaks at approximately 1 to 10 ms reflecting the accumulations of O. The concentration changes of P4 and P5 are highlighted in *red* because these states contribute to the accumulation of O at any Na^+^ concentration.

The next concern is which P state contributes to O accumulation. If O can be divided into substrates, O accumulation is probably described by more than two P states. Thus, we compared the time courses of concentration changes of P states and the flash-induced absorbance change at 600 nm. The latter involves the absorbance change because of the formation and decay of O. As shown in Figure 3, O accumulations are described by P4 and P5 states at all Na^+^ concentrations. Thus, both states contain Na^+^-binding intermediates.

**Figure 4:**
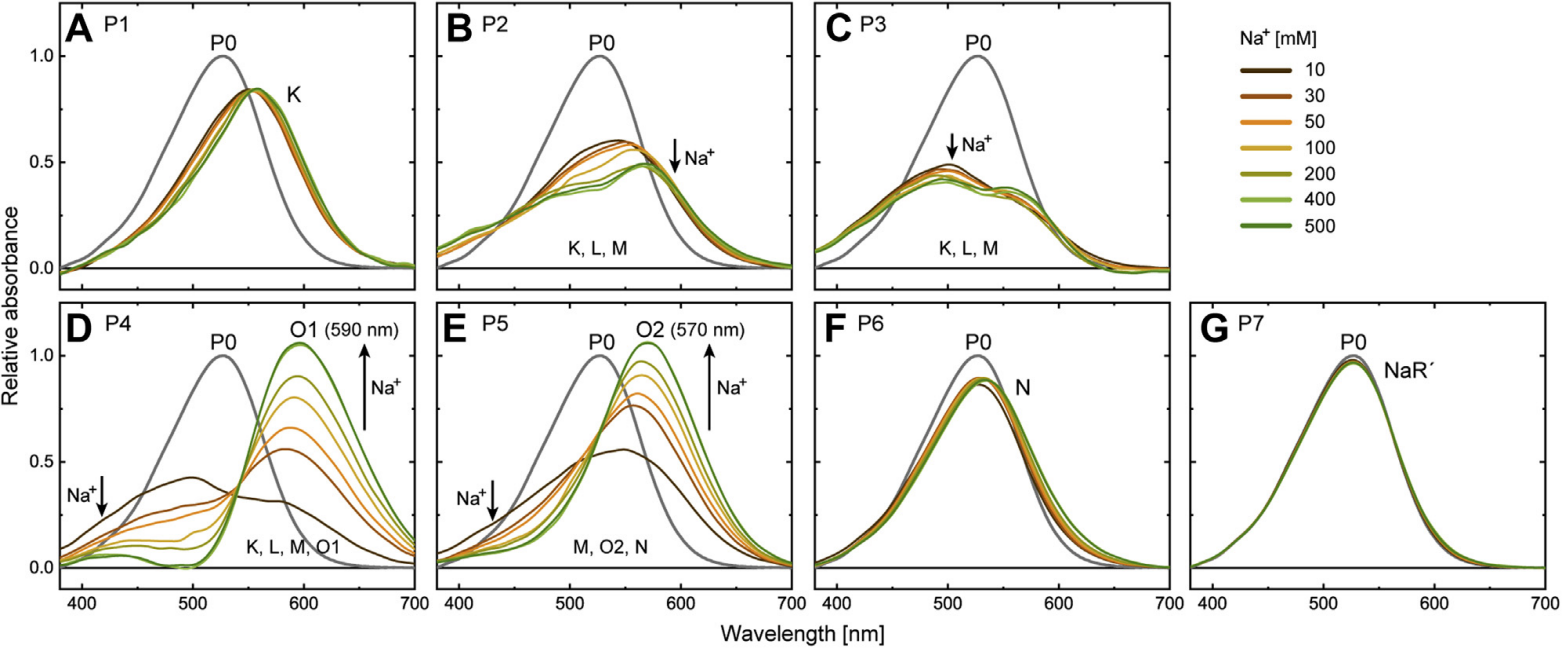
**Absorption spectra of the kinetically distinguishable Pi states (*i* = 1–7).** The spectra at seven Na^+^ concentrations (10, 30, 50, 100, 200, 400, and 500 mM) are plotted together in each panel. Based on the spectral analyses, we assigned the intermediates involved in respective Pi states (for details, see text). Their names are indicated in respective panels. P1 (*A*), P6 (*F*), and P7 (*G*) seem to contain single intermediates assigned to K, N, and NaR', respectively. Other states have broad spectra reflecting the quasi-equilibria of several intermediates. P2 (*B*) and P3 (*C*) were commonly assigned to mixtures of K, L, and M. These three intermediates also appear in P4 (*D*) together with the redshifted O1, whose λ_max_ is located at 590 nm. P5 (*E*) was assigned to the mixture of M, N, and another red-shifted O2, whose λ_max_ is located at 570 nm. Na^+^ concentration dependences appeared in the P2–P5 states. The *arrows* indicate these changes.

Next, we determined the absorption spectra of P states to probe the physical intermediates involved in each P state, especially in the P4 and P5 states. The results are summarized in Figure 4. As shown, both the P4 and P5 states contain red-shifted intermediates (Fig. 4, *D* and *E*) in equilibrium with the shorter wavelength intermediates. As the Na^+^ concentration is increased, the equilibria commonly shift toward red-shifted intermediates. Thus, these intermediates bind Na^+^ and correspond to the O intermediate. However, their λ_max_ values are clearly different. The O in P4 has λ_max_ at approximately 590 nm, whereas another O in P5 has a λ_max_ of approximately 570 nm. Thus, these intermediates should be assigned to different Na^+^-binding states. Hereafter, we call them O1 and O2, respectively.

### Physical intermediates involved in the kinetically defined states

**Figure 5:**
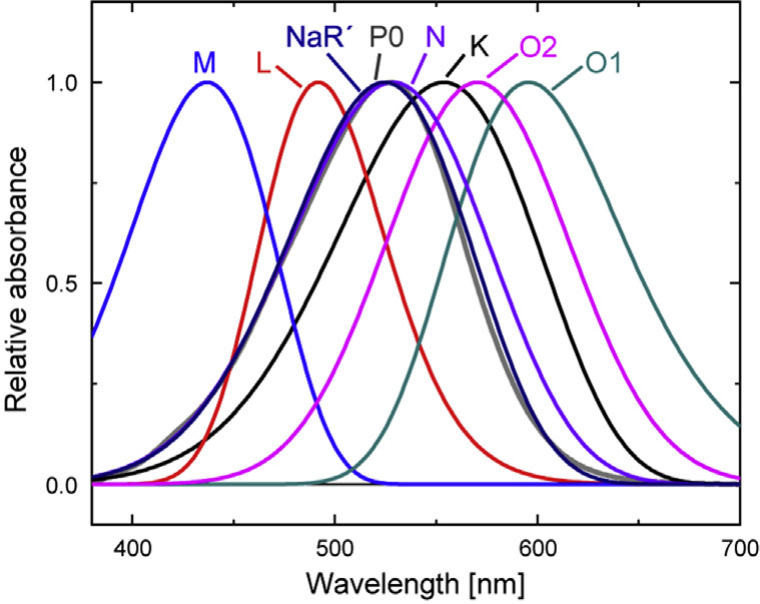
**Deduced absorption spectra of the physical intermediates.** All intermediate spectra were fitted by using SG functions, whose parameter values are summarized in Table S1. All spectra were normalized by their respective maximum amplitudes. P0 denotes the original dark state. SG, skewed Gaussian.

**Figure 6:**
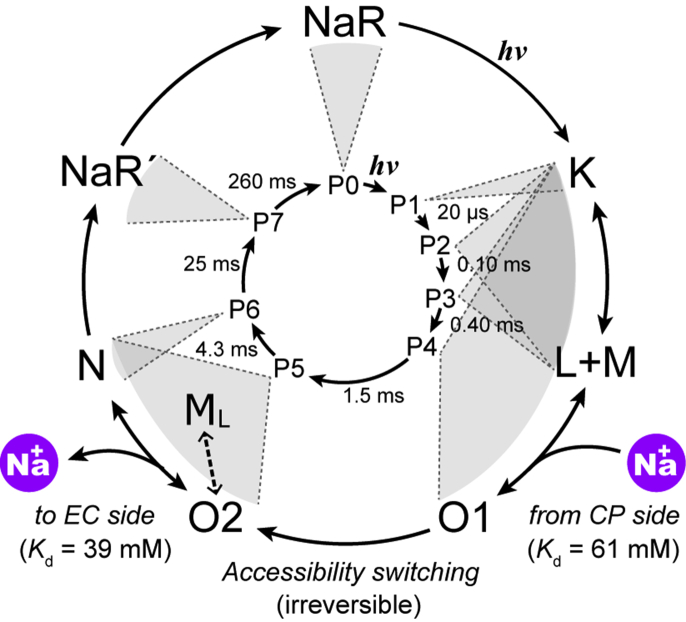
**Photocycle scheme deduced from this study.** The *inner circle* indicates the sequential irreversible model used for the global fitting analysis. Along this *circle*, the decay time constants at 200 mM Na^+^ are shown. The *outer circle* represents the deduced “true” photocycle scheme. The *gray circular sectors* indicate the intermediates involved in the respective Pi states. The Na^+^ uptake and release timings are also indicated with the deduced dissociation constant (*K*_*d*_) values for Na^+^. The O1 to O2 transition occurs irreversibly and corresponds to the process of accessibility switching. “L + M” indicates the simultaneous appearance of L and M, so their appearance order could not be determined. In addition to the P2–P4 states, the P5 state also contains M in a faint amount. This latter M is denoted with M_L_, which might be formed from O2 reflecting Na^+^ binding near the protonated Schiff base (for details, see text).

For further analysis, we deduced the absorption spectra of the physical intermediates. Here, we employed the skewed Gaussian (SG) function to simulate intermediate spectra (for details, see Experimental procedures section). The determined SG functions are plotted in Figure 5, and their parameters are listed in Table S1. As described later, we finally obtained the photocycle scheme shown in Figure 6, which involves the relationships between the P states and the physical intermediates.

### K, N, and NaR' intermediates

Of the seven P states, states P1, P6, and P7 (Fig. 4, *A*, *F*, and G) show single absorption bands independent of Na^+^ concentration. Thus, these states probably contain only a single intermediate. Indeed, they fitted well with a single SG function, as shown in Fig. S3. The first state, P1, should correspond to K. Meanwhile, the last state, P7, has almost the same spectrum as the dark state. Thus, P7 is a precursor of the dark state and should be named as NaR', which probably has almost the same protein conformation as the dark state. P6 contains a slightly red-shifted intermediate. We tentatively named this intermediate N. As discussed later, in the preceding P5 state, N and O2 probably attain Na^+^-dependent equilibrium. Thus, N seems to have a conformation whose Na^+^-binding site faces to the EC side. This conformation might be different from the subsequent NaR' and dark states. As mentioned previously, the dark state does not contain Na^+^. Thus, until the end of the photocycle, the binding site should become isolated from the medium.

### L, M, O1, and O2 intermediates

The P2 and P3 states have broad and similar spectra (Fig. 4, *B* and *C*). Thus, they seem to contain the same intermediates in the equilibria. Here, we assumed that K remains in the P2 and P3 states and confers longer absorptions. Fig. S4 shows approximations of the remaining spectra after subtracting the contribution of K. The remaining spectra are still broad, especially for P2, and thus probably contain two intermediates L and M. For H^+^-pump rhodopsins, the M intermediate is known to contain the deprotonated Schiff base and, thus, it exhibits the shortest absorption band among all intermediates. For M of NaR, this deprotonation was also experimentally confirmed (20). Similar to P2 and P3, a broad spectrum also appeared in P4 at 10 mM Na^+^ (Fig. 4*D*). Thus, P4 probably reflects the equilibrium between O1 and its preceding intermediates (K, L, and M), which should be associated with the Na^+^ uptake reaction at the CP side. Conversely, the P5 spectrum at 10 mM was relatively narrow (Fig. 4*E*) and close to that of N in the P6 state (Fig. 4*F*). Thus, P5 probably represents the equilibrium at the opposite side, where O2 is in equilibrium with the subsequent N. This equilibrium should be associated with the Na^+^ release reaction. The validity of this discussion was confirmed by following the decompositions of the P2–P5 spectra.

Here, we first analyzed the P4 and P5 spectra at 400 and 500 mM. In both states, the spectra at two Na^+^ concentrations were almost the same (Fig. 4, *D* and *E*). Thus, the shifts of the equilibria seem to be almost saturated above 400 mM. Fig. S5*A* supported this view. From the P4 and P5 spectra (Fig. 4, *D* and *E*), we picked up the absorbance values at λ_max_ of O1 (590 nm) and O2 (570 nm), respectively. These absorbance values are plotted in Fig. S5*A* against Na^+^ concentration. The increases in absorption values became slower at higher Na^+^ concentrations and almost saturated above 400 mM. Reflecting these changes, the P4 and P5 spectra above 400 mM Na^+^ appear to contain O1 and O2 as the respective main components, whereas at approximately 430 nm, both spectra also contain faint contributions from another intermediate. We tentatively assigned this intermediate as M, although its appearance in P5 seems unusual, as discussed later. We deduced the spectra of M, O1, and O2, as shown in Fig. S5*B*. Here, we first averaged the respective spectra at 400 and 500 mM Na^+^. The two resultant spectra were fitted simultaneously using the following equation:

(1) $$f_{\text{M}}\cdot {\text{SG}}_{\text{M}}+f_{\text{O}1}\cdot {\text{SG}}_{\text{O}1}+f_{\text{O}2}\cdot {\text{SG}}_{\text{O}2}$$

where SG_M_, SG_O1_, and SG_O2_ represent the SG functions for the respective absorption bands and are shared between two spectra in the fitting analysis. The terms *f*_M_, *f*_O1_, and *f*_O2_ are the coefficients to describe their respective amplitudes. As mentioned previously, O1 and O2 do not appear simultaneously in the P4 and P5 states. Thus, *f*_O2_ was fixed to zero for P4 fitting, whereas *f*_O1_ was fixed to zero for P5 fitting. The best-fit curves simulated the spectra well (Fig. S5*B*).

As described previously, P2 and P3 commonly contain K, L, and M. Thus, the spectra of P2 and P3 at all Na^+^ concentrations were fitted simultaneously by the following equation:

(2) $$f_{\text{K}}\cdot {\text{SG}}_{\text{K}}+f_{\text{L}}\cdot {\text{SG}}_{\text{L}}+f_{\text{M}}\cdot {\text{SG}}_{\text{M}}$$

**Figure 7:**
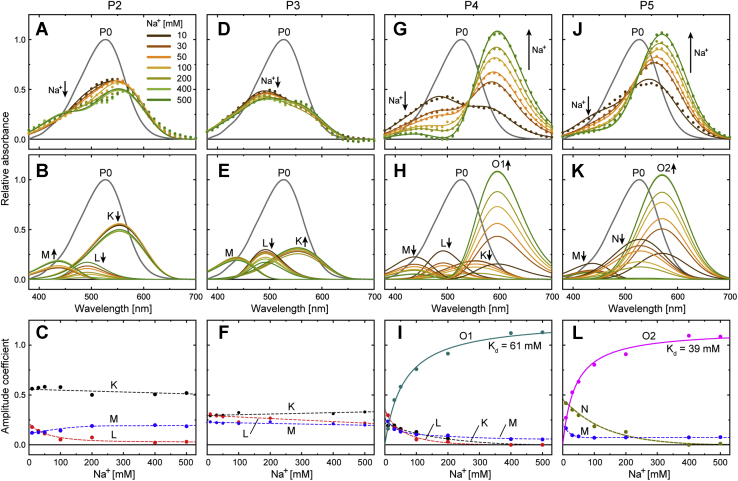
**Decompositions of the absorption spectra of P2–P5 states.** In the *top panelsA*, *D*, *G*, and *J*), the spectra of P states (*closed circles*) are plotted together with the results of fitting (*smooth lines*). The *middle panels* (*B*, *E*, *H*, and *K*) show the deduced spectra of physical intermediates, and their amplitude coefficients are plotted in the *bottom panels* (*C*, *F*, *I*, and *L*) against Na^+^ concentration. The *vertical arrows* in the *top and middle panels* indicate the spectral changes with increasing Na^+^ concentration. The amplitude coefficients of O1 (*panelI*) and O2 (*panelL*) were fitted with Equation 3, and their curves indicate the best-fit results. The determined parameter values (±standard errors) were *f*_max_ = 1.26 ± 0.03 and *K*_*d*_ = 60.6 ± 5.8 mM for O1 and *f*_max_ = 1.15 ± 0.04 and *K*_*d*_ = 38.8 ± 4.7 mM for O2.

Here, we assumed that SG_M_ is identical to that in Equation 1. Moreover, we determined SG_K_ from the analysis of P1 (Fig. S3*A*). Thus, in the fitting using Equation 2, we determined SG_L_ and the three amplitude coefficients, *f*_K_, *f*_L_, and *f*_M_. The best-fit results are summarized in the two left columns in Figure 7. The P2 spectra show a distinct Na^+^ concentration dependence (Fig. 7*A*). As shown in the lower panels (Fig. 7, *B* and *C*), this dependence reflects the decreases in the K and L fractions and an increase in the M fraction with increasing Na^+^ concentration. As mentioned previously, we performed time-resolved detection of Na^+^ concentration changes because of Na^+^ uptake and release by photolyzed NaR (11). Consequently, we detected the Na^+^-concentration change at only the formation and decay of O, indicating that no uptake and release of Na^+^ should occur until the formation of O. Thus, the Na^+^ dependence in P2 might originate from the binding of Na^+^ in the unphotolyzed state. The interface between NaR monomers is known to hold Na^+^ (21, 22). This binding in the dark state is not essential but affects the protein conformation and thermal stability (8, 13, 14, 23, 24, 25). This Na^+^ binding might also affect the equilibria in P2, although the reported dissociation constant (10–50 mM) (8, 25) seems to be somewhat smaller than that observed in P2 (Fig. 7*C*). The P3 spectra also show a small Na^+^-concentration dependence (Fig. 7*D*), which reflects the decrease in the L fraction and concomitant increase in the K fraction (Fig. 7, *E* and *F*). These changes might also originate from the Na^+^ binding in the dark state. However, this binding seems to be weak. Thus, there might exit another site to bind Na^+^. Such a binding site was also suggested in a previous study (26). As described previously, L and M appear simultaneously in P2 and remain together until P4. Thus, they are shown as “L + M” in Figure 6, reflecting the difficulty in determining the order of their appearance.

### Na^+^-concentration dependence of P4 and P5 states

The P4 and P5 spectra at lower Na^+^ concentrations (10–200 mM) remain to be analyzed. For P4 spectra, we first tried to fit them by assuming the involvement of M, L, and O1. However, the sum of these three SG functions could not simulate the spectra well (Fig. S6*A*). Better fitting results were obtained by assuming an additional contribution of K (Fig. S6*B*). The fitting results for all Na^+^ concentrations are summarized in Figure 7, *G*–*I*, where the sum of four SG functions (SG_K_, SG_L_, SG_M_, and SG_O1_) was adopted. For the P5 state, N is probably involved at low Na^+^ concentrations, as mentioned previously. Thus, we tried to fit the P5 spectra by assuming the involvement of M, O2, and N. As a result, well-fitted curves were obtained, as summarized in Figure 7, *J*–*L*. In both fitting analyses for P4 and P5 spectra, we determined only the amplitude coefficients of the respective intermediates (Fig. 7, *I* and *L*) because we had already determined all SG functions for the intermediates.

As described previously, the P4 spectra were fitted well with the contributions of O1 and the preceding intermediates of K, L, and M. Thus, O1 is probably accessible to the CP side and attains equilibrium with the preceding intermediates reflecting Na^+^ uptake at the CP side. Consistently, the amplitude coefficient of O1 is increased by the elevation of Na^+^ concentration, whereas the other coefficients decrease, as shown in Figure 7*I*. Conversely, to describe the P5 spectra, we had to consider the contribution of M in addition to O2 and N. However, the main components are O2 and N. Thus, P5 is probably a “counter state” of P4; in P5, O2 is probably accessible to the EC side and attains equilibrium with the subsequent N, reflecting the release of Na^+^ at the EC side. Their amplitude coefficients also show consistent changes (Fig. 7*L*); the value for O2 is increased, whereas the value for N is decreased by the elevation of Na^+^ concentration.

As mentioned previously, the appearance of M in P5 seems unusual. M is the intermediate before the formation of O1, and O1 is not involved in P5. Thus, M should not appear in P5, which should contain the intermediates after O1. The amplitude coefficient of M is also somewhat unusual because this coefficient is almost constant even with varying Na^+^ concentration (Fig. 7*L*). Thus, M in P5 might be a different intermediate from M in P2–P4. Hereafter, we use “M_L_” to denote the “late” M involved in P5. For “early” M in P2–P4, a vital role is considered for Na^+^ passage over the Schiff base region. At M, the Schiff base is deprotonated and thus seems to allow Na^+^ passage (10, 23). At subsequent O1 and O2, the Schiff base should be protonated and results in red-shifted spectra. Conversely, upon M_L_ formation, the Schiff base should deprotonate again, similar to the early M. We infer that this deprotonation might be a side reaction after Na^+^ binding near the protonated Schiff base. At O2, Na^+^ binding might lower the p*K*a of the Schiff base and subsequently induce deprotonation. Reflecting this view, M_L_ is shown in Figure 6 as an intermediate, which is not involved in the main pathway and formed from O2 as the branching reaction.

The amplitude coefficients are proportional to the respective mole fractions. Thus, we determined the dissociation constants (*K*_*d*_) of O1 and O2 for Na^+^. Here, we fitted their amplitude coefficients (Fig. 7, *I* and *L*) using the following equation:

(3) $$f_{\text{O}}=f_{\text{max}}\cdot \left[{\text{Na}}^{+}\right]/\left(K_{d}+\left[{\text{Na}}^{+}\right]\right)$$

where *f*_max_ and [Na^+^] denote the maximum amplitude coefficient and Na^+^ concentration, respectively. The best-fit curves shown in Figure 7, *I* and *L* were obtained at *K*_*d*_ values of 61 mM for O1 and 39 mM for O2.

For ideal transporters, the substrate affinities should be strong at the uptake side but weak at the release side. In contrast, NaR has a relatively strong affinity at the release side compared with the uptake side. This disadvantage might be overcome by the irreversible transition from O1 to O2. As shown previously, these intermediates do not appear in the same P state, indicating that equilibrium is not achieved. Thus, the O1 to O2 transition seems to be irreversible, which might assure one-way Na^+^ transfer even in transport against an electrochemical gradient across the membrane. The mechanism for the irreversible transition is an interesting subject to be solved in future studies. In addition to O1 → O2, the transitions after P5 also seem to be important. At the P5 state, N attains Na^+^-dependent equilibrium with O2. However, at the next P6 state, N solely appears at any Na^+^ concentration, implying that the Na^+^-binding site is isolated from the EC medium. Thus, the N in P6 might have a different conformation from the N in P5. This conformational change might also be a critical process for Na^+^ transport, even at a stronger affinity in the P5 state. Moreover, two other irreversible transitions, N → NaR' (P6 → P7) and NaR' → NaR (P7 → P0), probably assure the completion of the translocation cycle and contribute to one-way Na^+^ transport. The molecular events during these transitions should be clarified in future investigations.

## Conclusion

In this study, we analyzed the details of the NaR photocycle by using flash-induced absorbance changes. Consequently, O1 and O2 were identified as Na^+^-binding intermediates. They appear immediately after Na^+^ uptake and just before Na^+^ release. Thus, “accessibility switching” should occur during the O1 to O2 transition. To drive active transport, the switch process should involve strategic conformational changes, whose details should be clarified in future investigations.

The Na^+^-binding sites at O1 and O2 need to be identified. As mentioned previously, both intermediates commonly bind Na^+^ near the protonated Schiff base. The possible sites might be the vicinities of Asp115 and Asp250 residues, which correspond to Asp116 and Asp251, respectively, in *Krokinobacter eikastus* rhodopsin 2 (KR2), which is the first identified NaR (7, 8) and has a close similarity with the NaR (identity, 67%; similarity, 81%) used in this study. At present, KR2 is the only NaR whose structure has been solved (21, 23, 27, 28). The positions of the Asp residues in KR2 are indicated in Fig. S7*A*. Both Asp residues are known to be deprotonated in the dark state (8, 14, 21, 23, 29, 30) and play essential roles during the photocycle because their replacement with dissociable residues completely removes Na^+^-pump activity (8). It is not necessary to consider different positions for the Na^+^-binding site at O1 and O2. However, different positions seem to be rational to achieve accessibility switching during the O1 to O2 transition. Recently, two research groups independently reported O intermediate structures of KR2 (27, 28). Both structures contain Na^+^ inside the protein, but the positions are different: Na^+^ binds near Asp116 in one structure (27) (Fig. S7*B*, *left*) but near Asp251 in the other (28) (Fig. S7*B*, *right*). Thus, these structures appear to be associated with O1 and O2. However, the retinal conformations are puzzling: the former structure contains a distorted all-*trans* retinal, indicating the near completion of reisomerization, whereas the latter still contains the 13-*cis* retinal, although the latter Na^+^-binding site is closer to the exit side (EC side). Thus, the two O structures do not seem to correspond to O1 and O2 directly. Further studies are necessary to clarify the molecular details of these Na^+^-binding intermediates.

## Experimental procedures

### Preparation of the nanodisc containing NaR

This study used NaR from the eubacterium *Indibacter alkaliphilus*, whose expression plasmid for *Escherichia coli* has been reported previously (12). Expression and purification were performed as previously described (31). Briefly, NaR, having a C-terminal histidine tag, was expressed in *E. coli* BL21 cells. After solubilization with the detergent *n*-dodecyl-β-D-maltoside, the proteins were purified using a nickel–nitrilotriacetic acid agarose column. The NaR concentration was determined by using a molar extinction coefficient of 50,000 M^−1^ cm^−1^ at 527 nm. The purified sample was stored at 4 °C after replacing the buffer solution with 50 mM Tris–HCl, pH 8.0, 0.15 M NaCl, and 0.05% *n*-dodecyl-β-D-maltoside.

NaR incorporated into nanodiscs was used for all experiments. Here, we used MSP1E3D1 as the membrane scaffold protein (MSP), prepared as previously described (32). Briefly, the MSP encoded in the pET28a plasmid (Addgene plasmid #20066) was expressed in *E. coli* BL21(DE3) cells. The resultant MSP had an N-terminal histidine tag and was thus purified using a nickel–nitrilotriacetic acid agarose column. Purified MSP was stored at −30 °C after replacing the buffer solution with 10 mM Tris–HCl, pH 7.6, containing 0.1 M NaCl and 1 mM EDTA. The MSP concentration was calculated using ε_280_ = 29,900 M^−1^ cm^−1^.

Purified NaR was reconstituted into nanodiscs according to standard protocols, with some modifications (33). First, the MSP was mixed with *E. coli* lipid (Avanti), which was initially suspended in 25 mM cholic acid. After 20 min of incubation on ice, NaR was added to the mixture, further incubated for 20 min. The resultant mixture contained three components, that is, NaR, MSP, and lipids, at molar ratios of 1:1:130. Finally, the detergents were removed at 4 °C for 12 h by the addition of ~300 mg SM2 adsorbent Bio-Beads (Bio-Rad) per milliliter of solution.

### Flash photolysis spectroscopy

Flash-induced absorbance changes were measured in the −40 ms to 10 s time range on a single-wavelength kinetic system. At time 0, NaR was photoactivated by the second harmonic (7 ns, 532 nm) of a Q-switched neodymium-doped yttrium aluminum garnet laser (Surelite I-10; Continuum). The details of the setup have been previously described (16, 17). The data after 10 μs were used for the fitting analysis to avoid contamination of the laser scattering artifact, and the data before time 0 were used to calculate the baseline. Thirty laser pulses were applied at each measuring wavelength to improve the signal-to-noise ratio. The NaR nanodisc was suspended in 50 mM Tris–HCl (pH 8) containing various concentrations of NaCl and *N*-methyl-d-glucamine, an organic cation that is often used to replace Na^+^ in the sample. The buffer solutions were prepared by mixing two Tris–HCl (pH 8) buffer solutions containing 500 mM NaCl or 500 mM *N*-methyl-d-glucamine. For all measurements, the sample temperature was kept at 25 °C, and the NaR concentration was adjusted to approximately 10 μM.

The datasets measured at 380 to 700 nm with a 10 nm interval were analyzed based on an irreversible sequential model, which describes the photocycle by one-way transitions of kinetically distinguishable state Pi (*i* = 1, 2 … n) (15). The original unphotolyzed state was denoted by P0. This model contains only forward reactions. Thus, the Pi states may contain a few physically defined intermediates when reverse reactions exist between them. The steps for the analysis have been described previously (18, 34). The noise levels at each measuring wavelength were first evaluated by calculating the standard deviations of the data in the −40 to 0 ms time range. Reciprocals of the standard deviations were used as weights to calculate the fitting residuals at each wavelength. Thus, the standard deviations of the residuals should become 1 at any wavelength when the optimal fitting results are obtained. Here, a dataset of all wavelengths was simultaneously fitted by multiexponential functions. The number of exponents corresponds to that of the P states. This number was determined from the reduction in the standard deviation of the residuals, which was calculated for the entire dataset. This standard deviation should also be close to 1 if the best fitting results are obtained. After the fitting procedures, the difference in spectra between Pi and P0 (Δεi) was calculated using the determined parameters. Independently, the P0 spectrum was calculated from the absorption spectrum of the dark state. This procedure is described in the Supporting information. Here, we extracted the main absorption band around 527 nm and used it as the P0 spectrum. Finally, the absolute spectra of Pi states were obtained by adding the spectrum of P0 to the difference spectra, Δεi.

The absorption spectra of the physical intermediates were estimated using the SG function:

(4) $$A\left(\lambda \right)=\left\{ \begin{array}{l} \exp\left\{-\frac{\ln\;2}{{\left(\ln\;\rho \right)}^{2}}{\left[\ln\left(\frac{\left(1/\lambda -1/\lambda_{\text{max}}\right)\left(\rho^{2}-1\right)}{\Delta \nu \cdot \rho }+1\right)\right]}^{2}\right\},\;\text{if }\;\frac{1}{\lambda }>\frac{1}{\lambda_{\text{max}}}-\frac{\Delta \nu \cdot \rho }{\left(\rho^{2}-1\right)}\\ 0,\text{if}\;\frac{1}{\lambda }\leq \frac{1}{\lambda_{\text{max}}}-\frac{\Delta \nu \cdot \rho }{\left(\rho^{2}-1\right)} \end{array}\right.$$

which is defined by the three parameters: λ_max_, the wavelength at maximum absorbance, *ρ*, the skewness of the band, and Δ*ν*, the full width at half-maximum in wave number. This SG function has an amplitude of 1. Thus, the amplitude coefficient was introduced to describe the spectra involved in the P state.

## Data availability

All the data supporting the findings of this study are available within the article and the supporting information.

## Supporting information

This article contains supporting information.

## Conflict of interest

The authors declare that they have no conflicts of interest with the contents of this article.
