# Supplementary material for: Real-time identification of two substrate-binding intermediates for the light-driven sodium pump rhodopsin
Source: J Biol Chem. 2021 May 18;296:100792. doi: 10.1016/j.jbc.2021.100792 (PMC8219890; doi:10.1016/j.jbc.2021.100792)
Supplement: Supplemental Figures S1–S7 and Table S1 [file mmc1.pdf]

Supporting Information

Real-Time Identification of Two Substrate-Binding Intermediates for the Light-Driven Sodium-Pump Rhodopsin

Tomoya Kato,[†] Takashi Tsukamoto,[‡,§] Makoto Demura,[‡,§] and Takashi Kikukawa[*,‡,§]

[†]Graduate School of Life Science, Hokkaido University, Sapporo 060-0810, Japan

[‡]Faculty of Advanced Life Science, Hokkaido University, Sapporo, 060-0810, Japan

[§]Global Station for Soft Matter, Global Institution for Collaborative Research and Education, Hokkaido University, Sapporo, 001-0021, Japan

*To whom correspondence should be addressed.

Takashi Kikukawa

Email: kikukawa@sci.hokudai.ac.jp

Table of Contents

Supplemental Experimental Procedures S3

Determination of the P0 spectrum S3

Supplemental Figures and Tables S4

Figure S1. Determination of the P0 spectrum.................... S4

Figure S2. Flash-induced absorbance changes of NaR in the absence of Na^+.................... S5

Figure S3. Results of fitting for P1, P6 and P7 spectra with single SG functions.................... S6

Figure S4. Estimation of the physical intermediates involved in P2 and P3 states.................... S7

Figure S5. Decomposition of the absorption spectra of P4 and P5 states at high Na^+ concentrations.................... S8

Figure S6. Estimation of the physical intermediates involved in the P4 state at 10–200 mM Na^+.................... S9

Figure S7. NaR structures in the dark state and the O intermediate.................... S10

Table S1. Parameters of the SG functions to fit the intermediate spectra.................... S11

Supplemental Experimental Procedures

Determination of the P0 spectrum

The absorption spectrum of the P0 state was determined from the measured spectrum of the dark state and used for the spectral calculations of the Pi states. The spectrum of the dark state showed only negligible dependence on Na^+ concentration (data not shown). Here, we used the P0 spectrum at 400 mM Na^+ to calculate all spectra of Pi states. The procedures to determine the P0 spectrum are shown in Fig. S1. In addition to the scattering background, the measured spectrum (black line in the inset) includes three absorption bands: the main band at approximately 527 nm, the β-band at approximately 380 nm, and the band from the aromatic amino acid residues at approximately 280 nm. For the same analyses in previous reports (16-18), we used the sum of the two former bands as the P0 spectrum. However, the presence of a β-band is often confused with the appearance of short-wavelength intermediates in the Pi states. Here, we deduced the main band and used it as the P0 spectrum. First, we estimated the scattering background (green line in the inset) by the α / λ^4 (λ in nm) term. The value of α was determined to be 1.90×10^9. This scattering curve was subtracted from the measured spectrum. For the resultant spectrum (red line), we estimated the β-band (blue line) using the SG function in Eq. 4. The determined amplitude was 0.064, and the parameters for the SG function were λ_{max} = 383 nm, ρ = 1.20, and $\Delta\nu$ = 5357 cm^{-1}. Next, the main band (black line) was calculated by subtracting the β-band from the spectrum shown as the red line. Finally, the resultant band (black line) was normalized by its maximum amplitude and used as the P0 spectrum.

Supplemental Figures and Tables

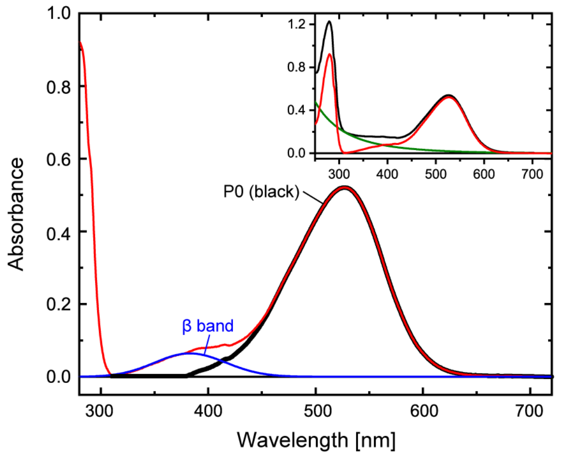

Figure S1. Determination of the P0 spectrum. The main absorption band around 527 nm (thick black line) was calculated and then used as the P0 spectrum. The measured absorption spectrum of the dark state is shown in the inset with a black line. First, the scattering background (green line) was estimated and then subtracted from the measured spectrum. The resultant spectrum is shown with red line. The P0 spectrum was obtained by subtracting the estimated β-band (blue line) from the red spectrum.

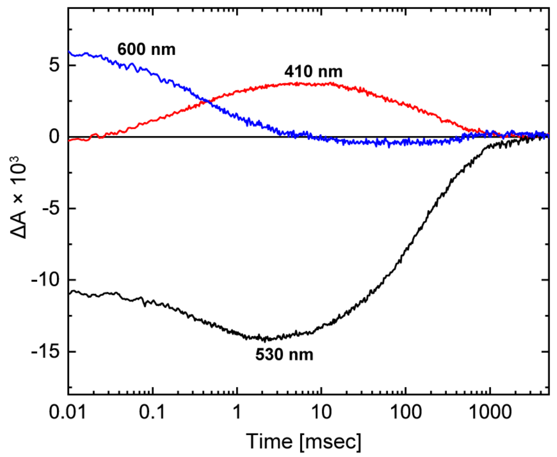

Figure S2. Flash-induced absorbance changes of NaR in the absence of Na^+. Other experimental conditions were the same as in Fig. 1C. Three traces were measured at 530 nm (black), 410 nm (red) and 600 nm (blue), respectively. The O intermediate was not formed in this condition.

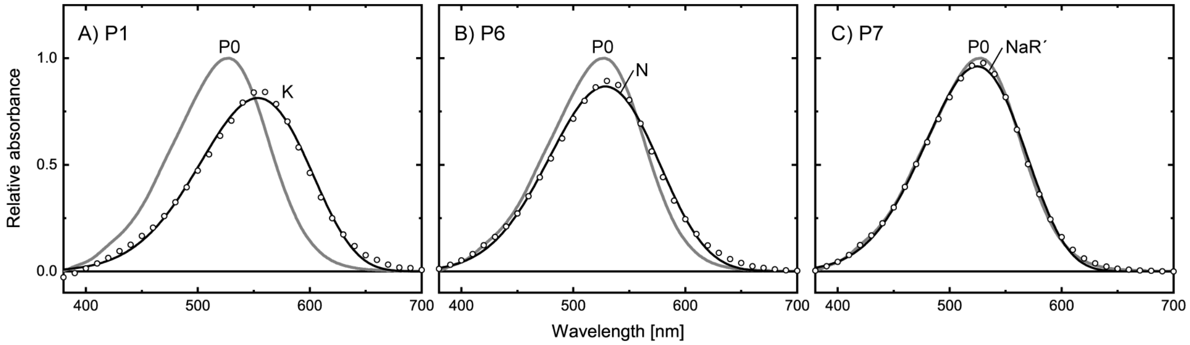

Figure S3. Results of fitting for P1, P6 and P7 spectra with single SG functions. The respective P spectra at the seven Na^+ concentrations were averaged and plotted with open circles. The smooth lines indicate the single SG functions providing the best-fit results. The determined SG functions are plotted in Fig. 5, and the parameters are listed in Table S1. The physical intermediate in P1 was assigned to K, and those in P6 and P7 were named N and NaR´, respectively.

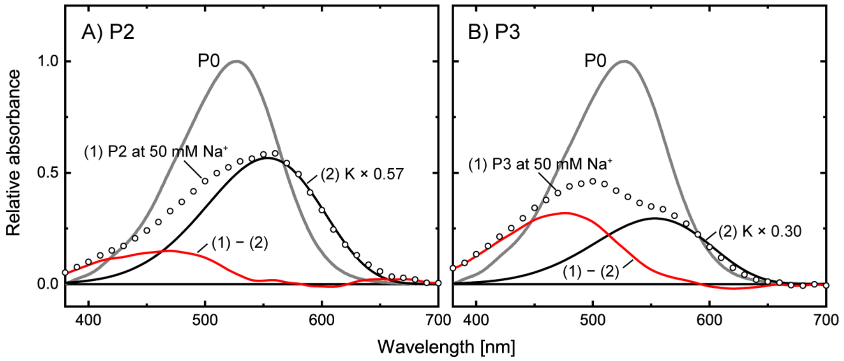

Figure S4. Estimation of the physical intermediates involved in P2 and P3 states. The P spectra at 50 mM Na^+ are plotted with open circles. Here, we assumed that the absorptions at long wavelength regions originate from the K intermediate. The estimated K components are shown with black lines calculated from the K in Fig. 5 by multiplying by 0.57 for P2 (Panel A) and 0.30 for P3 (Panel B). These K components were subtracted from the respective P spectra. The remaining spectra (red lines) are still broad and thus seem to originate from two physical intermediates of L and M.

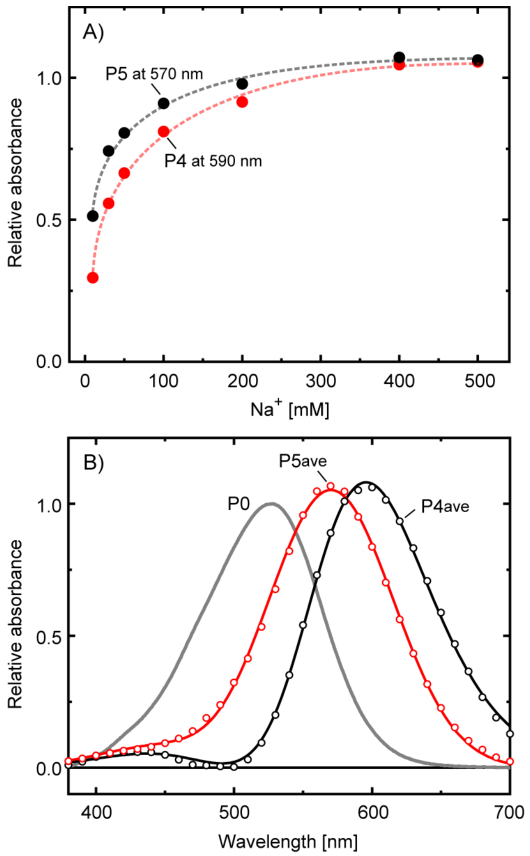

Figure S5. Decomposition of the absorption spectra of P4 and P5 states at high Na^+ concentrations. (A) Na^+ concentration dependences on the accumulations of O1 and O2 in the P4 and P5 states. From the absorption spectra of P4 and P5 states (Fig. 4D and E), the absorbance values at λ_{max} of O1 (590 nm) and O2 (570 nm) were picked up and plotted against Na^+ concentration. Both absorption values increased as the Na^+ concentration increased and then became almost constant above 400 mM Na^+. The broken lines are drawn to guide the eye. (B) Estimation of absorption spectra of M, O1, and O2 intermediates. As shown above and in Fig. 4D and E, the spectra of both P4 and P5 showed only negligible changes with increasing Na^+ concentration from 400 mM to 500 mM. Thus, they were first averaged, and the resultant spectra ($P4_{ave}$ and $P5_{ave}$, open circles) were fitted simultaneously by Eq. 1, where we assumed that $P4_{ave}$ involves M and O1, whereas $P5_{ave}$ involves M and O2. Smooth lines indicate the best-fit results. The determined SG functions and their parameters are shown in Fig. 5 and Table S1, respectively.

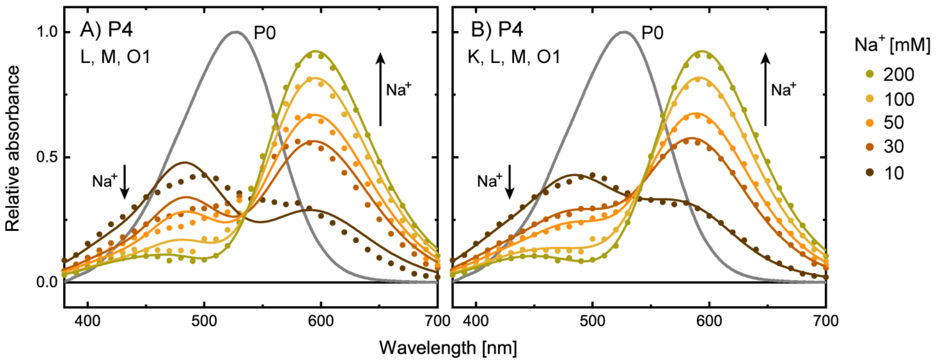

Figure S6. Estimation of the physical intermediates involved in the P4 state at 10–200 mM Na^+. The closed circles indicate the P4 spectra at 10, 30, 50, 100, and 200 mM Na^+. The smooth lines indicate the best-fit results by assuming the contributions of L, M, and O1 (A) and the additional contribution of K (B). For the SG functions, their parameters were fixed to those determined by the preceding analyses using Eqs. 1 and 2. The vertical arrows indicate the spectral changes with increasing Na^+ concentration.

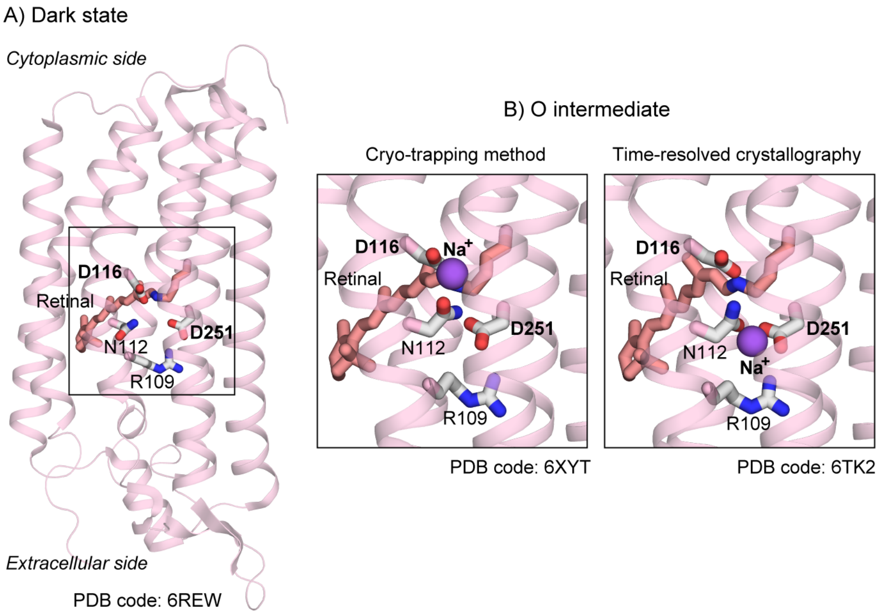

Figure S7. KR2 structures in the dark state and the O intermediate. At present, KR2 is only NaR, whose structures have been determined. Reflecting high amino acid identity, all residues shown in this figure are conserved in the NaR used in this study. Currently, two O intermediate structures of KR2 have been reported (27, 28). Their central regions are expanded and shown in the two right panels. The O structure obtained by the cryo-trapping method (Panel B, left) contains a distorted all-*trans* retinal and binds Na^+ near the Asp116 residue (PDB code: 6XYT). Conversely, another O structure was determined by time-resolved X-ray crystallography. This structure contains a 13-*cis* retinal and binds Na^+ near the Asp251 residue (PDB code: 6TK2).

Table S1. Parameters of the SG functions to fit the intermediate spectra

	λ_{max} [nm]	ρ	$\Delta\nu$ [cm^{-1}]
K	553.7 ± 1.0	1.469 ± 0.038	3910 ± 54
L	491.8 ± 0.9	0.981 ± 0.065	2997 ± 103
M	436.9 ± 5.5	1.496 ± 0.379	4558 ± 596
O1	595.6 ± 0.5	0.936 ± 0.014	2838 ± 20
O2	570.4 ± 0.5	1.194 ± 0.017	3272 ± 23
N	528.6 ± 0.8	1.370 ± 0.029	4194 ± 48
NaR´	524.6 ± 0.6	1.419 ± 0.023	3917 ± 34

The parameter values are shown with the standard deviations.
